# Tetra­aqua­bis(1-hydr­oxy-2-naphthoato-κ*O* ^2^)magnesium(II)

**DOI:** 10.1107/S1600536808006351

**Published:** 2008-03-14

**Authors:** Fu Huang, Wen-Dong Song

**Affiliations:** a College of Science, Guang Dong Ocean University, Zhanjiang 524088, People’s Republic of China

## Abstract

In the title mononuclear complex, [Mg(C_11_H_7_O_3_)_2_(H_2_O)_4_], the Mg^II^ atom is located on a centre of inversion and is coordinated by two O atoms from two 1-hydr­oxy-2-naphthoate ligands and four water mol­ecules in an octa­hedral geometry. The structure is consolidated by inter­molecular O—H⋯O hydrogen bonding, as well as by π–π stacking inter­actions between adjacent naphthyl ring systems [centroid–centroid distance between parallel naphthoate rings is 3.768 (2) Å].

## Related literature

For metal 1-hydr­oxy-2-naphthoates see: Ohki *et al.* (1986 ▶, 1987 ▶); Schmidt *et al.* (2005 ▶); Xue *et al.* (2005 ▶).
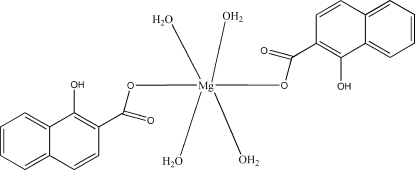


## Experimental

### 

#### Crystal data

[Mg(C_11_H_7_O_3_)_2_(H_2_O)_4_]
*M* *_r_* = 470.71
Monoclinic, 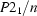

*a* = 6.7462 (5) Å
*b* = 5.2285 (4) Å
*c* = 29.965 (2) Å
β = 94.280 (5)°
*V* = 1054.01 (14) Å^3^
*Z* = 2
Mo *K*α radiation
μ = 0.14 mm^−1^
*T* = 296 (2) K
0.30 × 0.26 × 0.25 mm

#### Data collection

Bruker APEXII area-detector diffractometer
Absorption correction: multi-scan (*SADABS*; Sheldrick, 1996 ▶) *T*_min_ = 0.958, *T*_max_ = 0.965
8826 measured reflections
1936 independent reflections
1367 reflections with *I* > 2σ(*I*)
*R*_int_ = 0.040

#### Refinement

*R*[*F* ^2^ > 2σ(*F* ^2^)] = 0.046
*wR*(*F* ^2^) = 0.135
*S* = 1.06
1936 reflections
164 parameters
6 restraints
H atoms treated by a mixture of independent and constrained refinement
Δρ_max_ = 0.18 e Å^−3^
Δρ_min_ = −0.19 e Å^−3^

### 

Data collection: *APEX2* (Bruker, 2004 ▶); cell refinement: *SAINT* (Bruker, 2004 ▶); data reduction: *SAINT*; program(s) used to solve structure: *SHELXS97* (Sheldrick, 2008 ▶); program(s) used to refine structure: *SHELXL97* (Sheldrick, 2008 ▶); molecular graphics: *XP* in *SHELXTL* (Sheldrick, 2008 ▶); software used to prepare material for publication: *SHELXTL*.

## Supplementary Material

Crystal structure: contains datablocks I, global. DOI: 10.1107/S1600536808006351/ng2419sup1.cif

Structure factors: contains datablocks I. DOI: 10.1107/S1600536808006351/ng2419Isup2.hkl

Additional supplementary materials:  crystallographic information; 3D view; checkCIF report

## Figures and Tables

**Table 1:** Hydrogen-bond geometry (Å, °)

*D*—H⋯*A*	*D*—H	H⋯*A*	*D*⋯*A*	*D*—H⋯*A*
O2*W*—H3*W*⋯O1^i^	0.814 (9)	2.074 (13)	2.819 (2)	152 (2)
O2*W*—H4*W*⋯O3^ii^	0.821 (9)	1.888 (10)	2.698 (2)	169 (2)
O1*W*—H2*W*⋯O2*W*^iii^	0.813 (10)	2.136 (10)	2.924 (2)	163 (2)
O1*W*—H1*W*⋯O3	0.821 (9)	2.000 (13)	2.734 (2)	149 (2)
O1—H1⋯O2	0.82	1.75	2.4858 (19)	148

Symmetry codes: (i) 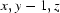; (ii) 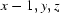; (iii) 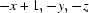.

**Fig. 1.**
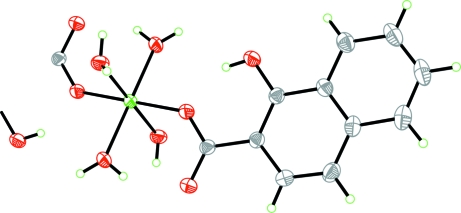
The structure of (I), showing the atomic numbering scheme. Non-H atoms are shown with 30% probability displacement ellipsoids. Unlabeled atoms are related to the labelled atoms by the symmetry operator (1 - x, 1 - y, -z).

**Fig. 2.**
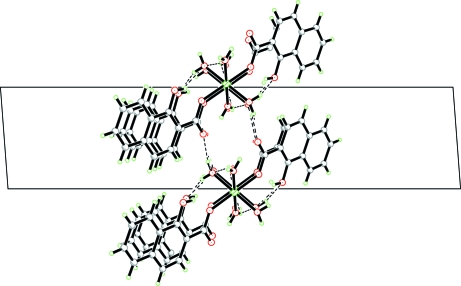
A packing view of the title compound. The intermolecluar hydrogen bonds are shown with dashed lines.

## supplementary crystallographic information

### Comment

In the structural investigation of 1-hydroxy-2-naphthoate complexes, it has been found that the 1-hydroxy-2-naphthoate functions as a multidentate ligand (Ohki *et al.*, 1986; 1987; Schmidt *et al.* (2005); Xue *et al.* (2005), with versatile binding and coordination modes. In this paper, we report the crystal structure of the title compound, (I), a new Mg complex obtained by the reaction of 1-naphthol-2-carboxylic acid with magnesium chloride in alkaline aqueous solution.

As illustrated in Figure 1, the Mg^II^ atom, lies on a centre of inversion, has an octahedral geometry, which is defined by two O atoms from two 1-hydroxy-2-naphthoate ligands and four water molecules (Fig. 1). The structural components are governed by intermolecular O—H···O hydrogen bond (Table 1) involving the coordinated water molecules, the hydroxy and carboxyl O atoms of 1-hydroxy-2-naphthoate ligands, and *via*π-π stacking interaction. The centroid to centroid distance between parallel naphthoate rings of neighboring complexes (at *x*, 1 + *y*, *z*) is 3.768 (2) Å, thus indicating a weak π-π stacking interaction.

### Experimental

A mixture of magnesium chloride(1 mmol), 1-hydroxy-2-naphthoate (1 mmol) NaOH (1.5 mmol) and H_2_O (12 ml) was placed in a 23 ml Teflon reactor, which was heated to 433 K for three days and then cooled to room temperature at a rate of 10 K h^-1^. The crystals obtained were washed with water and dryed in air.

### Refinement

Carbon-bound and hydroxyl H atoms were placed at calculated positions and were treated as riding on the parent C atoms with C—H = 0.93 Å, O—H = 0.82 Å and with *U*_iso_(H) = 1.2 *U*_eq_(C, O). Water H atoms were tentatively located in difference Fourier maps and were refined with distance restraints of O–H = 0.82 Å and H···H = 1.29 Å, each within a standard deviation of 0.01 Å, and with *U*_iso_(H) = 1.5 *U*_eq_(O).

### Crystal data

**Table:**

[Mg(C_11_H_7_O_3_)_2_(H_2_O)_4_]	*F*_000_ = 492
*M**_r_* = 470.71	*D*_x_ = 1.483 Mg m^−^^3^
Monoclinic, *P*2_1_/*n*	Mo *K*α radiation λ = 0.71073 Å
Hall symbol: -P 2yn	Cell parameters from 3500 reflections
*a* = 6.7462 (5) Å	θ = 1.3–26º
*b* = 5.2285 (4) Å	µ = 0.14 mm^−^^1^
*c* = 29.965 (2) Å	*T* = 296 (2) K
β = 94.280 (5)º	Block, colorless
*V* = 1054.01 (14) Å^3^	0.30 × 0.26 × 0.25 mm
*Z* = 2	

### Data collection

**Table:**

Bruker APEXII area-detector diffractometer	1936 independent reflections
Radiation source: fine-focus sealed tube	1367 reflections with *I* > 2σ(*I*)
Monochromator: graphite	*R*_int_ = 0.040
*T* = 296(2) K	θ_max_ = 25.5º
φ and ω scans	θ_min_ = 2.7º
Absorption correction: multi-scan(SADABS; Sheldrick, 1996)	*h* = −7→8
*T*_min_ = 0.958, *T*_max_ = 0.965	*k* = −6→6
8826 measured reflections	*l* = −36→28

### Refinement

**Table:**

Refinement on *F*^2^	Secondary atom site location: difference Fourier map
Least-squares matrix: full	Hydrogen site location: inferred from neighbouring sites
*R*[*F*^2^ > 2σ(*F*^2^)] = 0.046	H atoms treated by a mixture of independent and constrained refinement
*wR*(*F*^2^) = 0.135	*w* = 1/[σ^2^(*F*_o_^2^) + (0.0735*P*)^2^] where *P* = (*F*_o_^2^ + 2*F*_c_^2^)/3
*S* = 1.07	(Δ/σ)_max_ = 0.001
1936 reflections	Δρ_max_ = 0.18 e Å^−^^3^
164 parameters	Δρ_min_ = −0.19 e Å^−^^3^
6 restraints	Extinction correction: none
Primary atom site location: structure-invariant direct methods	

### Special details

**Table:**

Geometry. All e.s.d.'s (except the e.s.d. in the dihedral angle between two l.s. planes) are estimated using the full covariance matrix. The cell e.s.d.'s are taken into account individually in the estimation of e.s.d.'s in distances, angles and torsion angles; correlations between e.s.d.'s in cell parameters are only used when they are defined by crystal symmetry. An approximate (isotropic) treatment of cell e.s.d.'s is used for estimating e.s.d.'s involving l.s. planes.
Refinement. Refinement of *F*^2^ against ALL reflections. The weighted *R*-factor *wR* and goodness of fit *S* are based on *F*^2^, conventional *R*-factors *R* are based on *F*, with *F* set to zero for negative *F*^2^. The threshold expression of *F*^2^ > σ(*F*^2^) is used only for calculating *R*-factors(gt) *etc*. and is not relevant to the choice of reflections for refinement. *R*-factors based on *F*^2^ are statistically about twice as large as those based on *F*, and *R*- factors based on ALL data will be even larger.

### Fractional atomic coordinates and isotropic or equivalent isotropic displacement parameters (Å^2^)

**Table:**

	*x*	*y*	*z*	*U*_iso_*/*U*_eq_	
C1	0.8444 (3)	0.6423 (4)	0.06903 (7)	0.0358 (5)	
C2	0.9063 (3)	0.8351 (4)	0.10386 (7)	0.0357 (5)	
C3	0.7694 (3)	1.0058 (4)	0.11954 (7)	0.0364 (6)	
C4	0.8258 (4)	1.1864 (4)	0.15346 (7)	0.0395 (6)	
C5	0.6880 (4)	1.3630 (4)	0.16921 (8)	0.0509 (7)	
H5	0.5572	1.3641	0.1570	0.061*	
C6	0.7470 (5)	1.5322 (4)	0.20245 (9)	0.0608 (8)	
H6	0.6556	1.6466	0.2129	0.073*	
C7	0.9423 (5)	1.5343 (5)	0.22074 (9)	0.0666 (9)	
H7	0.9804	1.6506	0.2432	0.080*	
C8	1.0783 (5)	1.3682 (5)	0.20608 (9)	0.0613 (8)	
H8	1.2083	1.3720	0.2188	0.074*	
C9	1.0249 (4)	1.1889 (4)	0.17163 (7)	0.0463 (6)	
C10	1.1625 (4)	1.0146 (4)	0.15507 (8)	0.0515 (7)	
H10	1.2943	1.0166	0.1667	0.062*	
C11	1.1040 (3)	0.8449 (4)	0.12247 (8)	0.0451 (6)	
H11	1.1968	0.7320	0.1122	0.054*	
Mg1	0.5000	0.5000	0.0000	0.0354 (3)	
O1	0.5757 (2)	1.0040 (3)	0.10408 (5)	0.0463 (5)	
H1	0.5581	0.8945	0.0846	0.069*	
O2	0.6611 (2)	0.6510 (3)	0.05335 (5)	0.0439 (4)	
O3	0.9626 (2)	0.4811 (3)	0.05591 (5)	0.0467 (5)	
O1W	0.7019 (2)	0.2055 (3)	0.00065 (6)	0.0473 (5)	
H1W	0.806 (2)	0.238 (4)	0.0157 (8)	0.071*	
H2W	0.716 (4)	0.061 (3)	−0.0087 (8)	0.071*	
O2W	0.3269 (2)	0.2933 (3)	0.04349 (5)	0.0437 (5)	
H4W	0.221 (2)	0.351 (5)	0.0510 (7)	0.066*	
H3W	0.387 (3)	0.249 (5)	0.0668 (5)	0.066*	

### Atomic displacement parameters (Å^2^)

**Table:**

	*U*^11^	*U*^22^	*U*^33^	*U*^12^	*U*^13^	*U*^23^
C1	0.0323 (14)	0.0336 (11)	0.0417 (14)	−0.0011 (10)	0.0052 (11)	0.0022 (10)
C2	0.0348 (14)	0.0336 (12)	0.0384 (13)	−0.0021 (10)	0.0009 (10)	0.0018 (9)
C3	0.0360 (14)	0.0334 (11)	0.0396 (13)	−0.0016 (10)	0.0016 (11)	0.0019 (10)
C4	0.0485 (15)	0.0329 (11)	0.0382 (14)	−0.0028 (11)	0.0095 (11)	0.0016 (10)
C5	0.0627 (18)	0.0401 (13)	0.0513 (16)	−0.0034 (12)	0.0129 (13)	−0.0026 (11)
C6	0.088 (2)	0.0398 (14)	0.0573 (18)	−0.0046 (14)	0.0244 (17)	−0.0095 (12)
C7	0.098 (3)	0.0509 (16)	0.0522 (18)	−0.0182 (16)	0.0125 (18)	−0.0174 (13)
C8	0.071 (2)	0.0593 (16)	0.0522 (17)	−0.0175 (16)	−0.0047 (15)	−0.0068 (13)
C9	0.0549 (17)	0.0440 (13)	0.0399 (14)	−0.0125 (12)	0.0033 (12)	−0.0035 (11)
C10	0.0436 (16)	0.0592 (16)	0.0502 (16)	−0.0065 (12)	−0.0060 (12)	−0.0050 (12)
C11	0.0354 (15)	0.0487 (14)	0.0512 (16)	0.0009 (11)	0.0037 (12)	−0.0032 (11)
Mg1	0.0318 (6)	0.0267 (5)	0.0476 (7)	0.0029 (4)	0.0022 (5)	−0.0050 (4)
O1	0.0387 (11)	0.0452 (10)	0.0543 (11)	0.0098 (7)	−0.0017 (8)	−0.0129 (7)
O2	0.0330 (10)	0.0410 (9)	0.0564 (10)	0.0061 (7)	−0.0047 (8)	−0.0123 (7)
O3	0.0366 (10)	0.0454 (10)	0.0581 (11)	0.0067 (7)	0.0035 (8)	−0.0127 (7)
O1W	0.0399 (11)	0.0299 (8)	0.0711 (13)	0.0059 (7)	−0.0019 (9)	−0.0112 (8)
O2W	0.0360 (10)	0.0402 (9)	0.0554 (11)	0.0071 (7)	0.0068 (8)	0.0016 (8)

### Geometric parameters (Å, °)

**Table:**

C1—O3	1.244 (2)	C8—H8	0.9300
C1—O2	1.290 (2)	C9—C10	1.417 (3)
C1—C2	1.488 (3)	C10—C11	1.357 (3)
C2—C3	1.391 (3)	C10—H10	0.9300
C2—C11	1.407 (3)	C11—H11	0.9300
C3—O1	1.353 (3)	Mg1—O2^i^	2.0254 (15)
C3—C4	1.418 (3)	Mg1—O2	2.0254 (15)
C4—C9	1.411 (3)	Mg1—O1W	2.0551 (14)
C4—C5	1.416 (3)	Mg1—O1W^i^	2.0551 (14)
C5—C6	1.369 (3)	Mg1—O2W	2.1112 (15)
C5—H5	0.9300	Mg1—O2W^i^	2.1112 (15)
C6—C7	1.388 (4)	O1—H1	0.8200
C6—H6	0.9300	O1W—H1W	0.821 (9)
C7—C8	1.360 (4)	O1W—H2W	0.813 (10)
C7—H7	0.9300	O2W—H4W	0.821 (9)
C8—C9	1.421 (3)	O2W—H3W	0.814 (9)
			
O3—C1—O2	121.9 (2)	C11—C10—H10	119.7
O3—C1—C2	121.9 (2)	C9—C10—H10	119.7
O2—C1—C2	116.24 (19)	C10—C11—C2	121.7 (2)
C3—C2—C11	118.4 (2)	C10—C11—H11	119.2
C3—C2—C1	120.9 (2)	C2—C11—H11	119.2
C11—C2—C1	120.7 (2)	O2^i^—Mg1—O2	180.00 (6)
O1—C3—C2	121.89 (19)	O2^i^—Mg1—O1W	91.81 (6)
O1—C3—C4	116.78 (19)	O2—Mg1—O1W	88.19 (6)
C2—C3—C4	121.3 (2)	O2^i^—Mg1—O1W^i^	88.19 (6)
C9—C4—C5	119.7 (2)	O2—Mg1—O1W^i^	91.81 (6)
C9—C4—C3	118.6 (2)	O1W—Mg1—O1W^i^	180.00 (12)
C5—C4—C3	121.7 (2)	O2^i^—Mg1—O2W	89.92 (6)
C6—C5—C4	120.1 (3)	O2—Mg1—O2W	90.08 (6)
C6—C5—H5	120.0	O1W—Mg1—O2W	90.42 (6)
C4—C5—H5	120.0	O1W^i^—Mg1—O2W	89.58 (6)
C5—C6—C7	120.6 (3)	O2^i^—Mg1—O2W^i^	90.08 (6)
C5—C6—H6	119.7	O2—Mg1—O2W^i^	89.92 (6)
C7—C6—H6	119.7	O1W—Mg1—O2W^i^	89.58 (6)
C8—C7—C6	120.7 (2)	O1W^i^—Mg1—O2W^i^	90.42 (6)
C8—C7—H7	119.6	O2W—Mg1—O2W^i^	180.00 (10)
C6—C7—H7	119.6	C3—O1—H1	109.5
C7—C8—C9	121.0 (3)	C1—O2—Mg1	135.80 (13)
C7—C8—H8	119.5	Mg1—O1W—H1W	112.7 (16)
C9—C8—H8	119.5	Mg1—O1W—H2W	142.1 (16)
C4—C9—C10	119.3 (2)	H1W—O1W—H2W	105.2 (15)
C4—C9—C8	118.0 (2)	Mg1—O2W—H4W	120.8 (18)
C10—C9—C8	122.8 (3)	Mg1—O2W—H3W	114.4 (18)
C11—C10—C9	120.6 (2)	H4W—O2W—H3W	104.8 (15)

Symmetry codes: (i) −*x*+1, −*y*+1, −*z*.

### Hydrogen-bond geometry (Å, °)

**Table:**

*D*—H···*A*	*D*—H	H···*A*	*D*···*A*	*D*—H···*A*
O2W—H3W···O1^ii^	0.814 (9)	2.074 (13)	2.819 (2)	152 (2)
O2W—H4W···O3^iii^	0.821 (9)	1.888 (10)	2.698 (2)	169 (2)
O1W—H2W···O2W^iv^	0.813 (10)	2.136 (10)	2.924 (2)	163 (2)
O1W—H1W···O3	0.821 (9)	2.000 (13)	2.734 (2)	149 (2)
O1—H1···O2	0.82	1.75	2.4858 (19)	148

Symmetry codes: (ii) *x*, *y*−1, *z*; (iii) *x*−1, *y*, *z*; (iv) −*x*+1, −*y*, −*z*.

## References

[bb1] Bruker (2004). *APEX2* and *SAINT* Bruker AXS Inc., Madison, Wisconsin, USA.

[bb2] Ohki, Y., Suzuki, Y., Shimoi, M. & Ouchi, A. (1987). *Bull. Chem. Soc. Jpn*, **60**, 551–556.

[bb3] Ohki, Y., Suzuki, Y., Takeuchi, T., Shimoi, M. & Ouchi, A. (1986). *Bull. Chem. Soc. Jpn*, **60**, 1015–1019.

[bb4] Schmidt, M. U., Alig, E., Fink, L., Bolte, M., Panisch, R., Pashchenko, V., Wolf, B. & Lang, M. (2005). *Acta Cryst.* C**61**, m361–m364. 10.1107/S0108270105015659 15997067

[bb5] Sheldrick, G. M. (1996). *SADABS* University of Göttingen, Germany.

[bb6] Sheldrick, G. M. (2008). *Acta Cryst.* A**64**, 112–122. 10.1107/S0108767307043930 18156677

[bb7] Xue, Y. W., Xu, Q. F., Zhang, Y. & Lu, J. M. (2005). *Chin. J. Inorg. Chem.* **21**, 1735–1739.

